# What the papers say

**DOI:** 10.1093/jhps/hnad033

**Published:** 2023-10-18

**Authors:** Ali Bajwa

**Affiliations:** The Villar Bajwa Practice, London and Cambridge, UK

The *Journal of Hip Preservation Surgery* (*JHPS*) is not the only place where work in the field of hip preservation can be published. Although our aim is to offer the best of the best, we are continually fascinated by work, which finds its way into journals other than our own. There is much to learn from it, and so *JHPS* has selected six recent and topical subjects for those who seek a summary of what is taking place in our ever-fascinating world of hip preservation. What you see here are the mildly edited abstracts of the original articles, to give them what *JHPS* hopes is a more readable feel. If you are pushed for time, what follows should take you no more than 10 min to read. So here goes …

## HIGH-LEVEL COMPETITIVE ATHLETES WHO UNDERGO HIP ARTHROSCOPY DEMONSTRATE DURABLE 5-YEAR OUTCOMES AND LOWER SUBJECTIVE PAIN: A PROPENSITY-MATCHED ANALYSIS

The authors from Chicago [1] note that hip arthroscopy (HA) has been proven to be an effective treatment for femoroacetabular impingement syndrome (FAIS) in both competitive athletes (CAs) and non-CAs at short-term follow-up. However, there is a paucity of literature investigating mid-term outcomes comparing athletes with Controls. Their hypothesis was that the athletes would have significant improvements at 5 years, with favourable outcomes compared with their control counterparts, and high return-to-sport (RTS) rates. They used a propensity-matched retrospective comparative cohort study design.

CAs who underwent primary HA for FAIS from 1 January 2012 to 30 April 2017 were identified and propensity matched on a 1:4 basis to Controls by age, sex and body mass index (BMI). Patient-reported outcomes (PROs) were collected pre-operatively and at 5 years. Minimal clinically important difference (MCID) and patient-acceptable symptom states (PASS) rates were calculated using previously published thresholds. The rate and duration of RTS were collected retrospectively.

A total of 57 high-level CAs, 33 females and 24 males with a mean age of 21.7 years and a mean BMI of 23.1 kg/m^2^ were propensity matched to 228 Controls that included 132 females and 96 males with a mean age of 23.3 years and a mean BMI of 23.8 kg/m^2^. Significant differences were observed in pre-operative Hip Outcome Score Activities of Daily Living (HOS-ADL) subscales (CAs, 74.9 ± 13.7 versus Controls, 66.4 ± 18.4) and modified Harris Hip Score (mHHS) (CAs, 64.7 ± 12.9 versus Controls, 59.7 ± 14.3). Both groups demonstrated significant post-operative improvements in all outcome scores measured. At 5 years post-operatively, there were significant differences between groups in Visual Analogue Scale (VAS) for Pain (CAs, 17.3 ± 17.6 versus Controls, 24.7 ± 25.9). There were no significant differences in achieving MCID or PASS. Athletes RTS at a median of 25.2 weeks with an overall RTS rate of 90%. Similar rates of revision were seen between CA patients (*n* = 3; 5.3%) and Control patients (*n* = 9; 3.9%).

The authors concluded that CAs demonstrated significant and durable improvements in PROs as well as high MCID and PASS achievement rates after primary HA, which were comparable with those of Controls. Clinicians should be aware that CA patients demonstrate higher pre-operative mHHS and HOS-ADL scores than Controls and achieve lower average self-reported pain at 5 years post-operatively. In addition, CA patients demonstrate high rates of RTS at a median of 25 weeks post-operatively.

This study provided insight into CA versus Control PROs and rates of achieving MCID and PASS at a mid-term follow-up of 5 years. Furthermore, this study offers a perception of the RTS rate, both in general as well as specified to individualized sports.

## FIVE-YEAR OUTCOMES OF PRIMARY HIP ARTHROSCOPY FOR FEMOROACETABULAR IMPINGEMENT SYNDROME AMONG FEMALE PATIENTS: HIGHER BODY MASS INDEX IS ASSOCIATED WITH REDUCED CLINICALLY SIGNIFICANT OUTCOMES

The purpose of this study stated by Shankar *et al*. [2] was to evaluate the impact of age, BMI and symptom duration on 5-year clinical outcomes among females following primary HA for FAIS.

They conducted a retrospective review of a prospectively collected database of HA patients with a minimum 5-year follow-up. Patients were stratified by age (<30, 30–45 and ≥45 years), BMI (<25.0, 25.0–29.9 and ≥30.0) and pre-operative symptom duration (<1 versus ≥1 year). PROs were assessed using the mHHS and Non-arthritic Hip Score (NAHS). Pre- to post-operative improvement in mHHS and NAHS was compared between groups using the Mann–Whitney *U*-test or Kruskal–Wallis test. Hip survivorship rates and MCID achievement rates were compared with Fisher’s exact test. Predictors of outcomes were identified using multivariable linear and logistic regression.

They included 103 patients in the analysis with a mean age of 42.0 years (range 16–75) and a mean BMI of 24.9 (range 17.2–38.9). Most patients had symptoms of duration ≥1 year (60.2%). Six patients (5.8%) had arthroscopic revisions and two patients (1.9%) converted to total hip arthroplasty by a 5-year follow-up. Patients with BMI ≥30.0 had significantly lower post-operative mHHS (*P* = 0.03) and NAHS (*P* = 0.04) than those with BMI <25.0. Higher BMI was associated with reduced improvement in mHHS and lower odds of achieving the mHHS MCID (OR = 0.82) and NAHS MCID (OR = 0.88). Older age was predictive of reduced improvement in NAHS. Symptom duration ≥1 year was predictive of higher odds of achieving the NAHS MCID (OR = 3.98).

The authors concluded that female patients across a wide range of ages, BMIs and symptom durations experience satisfactory 5-year outcomes following primary HA, but higher BMI is associated with reduced improvement in PROs.

## TEN-YEAR SURVIVORSHIP, OUTCOMES, AND SPORTS PARTICIPATION IN ATHLETES AFTER PRIMARY HIP ARTHROSCOPY FOR FEMOROACETABULAR IMPINGEMENT SYNDROME

The authors from Chicago and Texas [3] note that HA is an effective treatment tool for athletes with FAIS. However, long-term data are scarce. They aimed to assess survivorship, minimum 10-year PRO measures (PROMs) and sports participation after primary HA for FAIS in athletes and to perform a propensity-matched comparison between patients undergoing labral debridement and labral repair in this cohort study.

Athletes who underwent HA for FAIS between February 2008 and December 2010 were eligible. The exclusion criteria were other ipsilateral hip conditions, Tönnis grade ≥2 or no baseline PROMs. Survivorship was defined as no conversion to total hip arthroplasty. The PASS, MCID, maximum outcome improvement (MOI) satisfaction threshold and sports participation were reported. A propensity-matched comparison between labral debridement and labral repair was performed. Two additional propensity-matched subanalyses were performed for capsular management and cartilage damage.

In total, 189 hips (177 patients) were included. The mean ± SD follow-up was 127.2 ± 6.0 months. Survivorship was 85.7%. Significant improvement in all PROMs was reported (*P* < 0.001). A total of 46 athletes with labral repair were propensity matched to 46 athletes with labral debridement. This subanalysis demonstrated significant and comparable improvement in all PROMs at a minimum 10-year follow-up (*P* < 0.001). For the labral repair group, the PASS achievement rates were 88.9% for the mHHS and 80% for the Hip Outcome Score of Sport-Specific Subscale (HOS-SSS); the MCID achievement rates were 80.6% for the mHHS and 84% for HOS-SSS; and for the MOI satisfaction threshold, rates were 77.8%, 80.6% and 55.6% for the mHHS, NAHS and VAS, respectively. For the labral debridement group, the PASS achievement rates were 85.3% for the mHHS and 70.4% for the HOS-SSS; the MCID achievement rates were 81.8% for the mHHS and 74.1% for HOS-SSS; and for the MOI satisfaction threshold, rates were 72.7%, 81.8% and 66.7% for the mHHS, NAHS, and VAS, respectively. Total hip arthroplasty conversions occurred significantly sooner with labral debridement than with labral repair (*P* = 0.048). Age was identified as a significant predictor of achieving the PASS.

The authors thus concluded that primary HA for FAI syndrome in athletes results in 85.7% survivorship and sustained PROM improvement at a minimum 10-year follow-up. A significant time delay to total hip arthroplasty conversion at a 10-year follow-up was reported with labral repair over debridement, although this should be interpreted with caution, as the total number of conversions was small.

## ACETABULAR LABRAL REPAIR AND SELECTIVE LABRAL DEBRIDEMENT SHOW NO SIGNIFICANT DIFFERENCE IN CLINICAL OUTCOMES AT A MINIMUM 2-YEAR FOLLOW-UP

In this study from multiple centres in China [4], the authors compare the outcomes of arthroscopic labral repair using looped-type sutures with a matched-pair selective labral debridement with a minimum 2-year follow-up.

They identified 378 patients undergoing primary arthroscopic labral repair using loop-suture and selective labral debridement from 2 January 2018 to 28 December 2020. The labral repair group was matched 1:1 to a selective labral debridement control group by age, sex, BMI, follow-up period, lateral centre-edge angle, Tönnis grade and pre-operative joint space. Before surgery, 3 Tesla (T) radial magnetic resonance imaging (MRI) with a 3D double-echo steady-state sequence was obtained following failed non-operative treatment lasting more than 3 months. Follow-up imaging was conducted at a minimum of 2 years. In both groups, the ratio of positive slices in which a disrupted chondrolabral junction was observed between the 2 o’clock and 11 o’clock positions was measured. PRO scores (PROs) included the HHS, VAS, HOS-ADL and HOS-SSS.

A total of 76 patients of the repair group were matched to 76 controls with a minimum 2-year follow-up. The repair group experienced a significant 2-fold improvement (0.6 ± 0.1 to 0.3 ± 0.1), while the selective debridement group experienced a significant 3-fold improvement (0.3 ± 0.1 to 0.1 ± 0.1). Significant improvement of the PROs was shown in both groups at a final follow-up without significant difference between the two groups.

The authors thus concluded that the mid-term clinical outcomes are comparable between the labral repair using looped-type sutures and the selective labral debridement group. Although a gap between the labrum and articular cartilage may appear in 3D DESS MRI results after labral repair, it does not correspond with clinical outcomes.

## SMARTPHONE TECHNOLOGY TO REMOTELY MEASURE POSTURAL SWAY DURING DOUBLE- AND SINGLE-LEG SQUATS IN ADULTS WITH FEMOROACETABULAR IMPINGEMENT AND THOSE WITH NO HIP PAIN

The authors from Australia [5] report that the COVID-19 pandemic has accelerated the demand for utilizing telehealth as a major mode of health-care delivery, with increasing interest in the use of teleplatforms for remote patient assessment. In this context, the use of smartphone technology to measure squat performance in people with and without FAIS has not been reported yet. They developed a novel smartphone application, the TelePhysio app, which allowed the clinician to remotely connect to the patient’s device and measure their squat performance in real time using the smartphone inertial sensors. The aim of this study was to investigate the association and test–retest reliability of the TelePhysio app in measuring postural sway performance during a double-leg (DLS) and single-leg (SLS) squat task. In addition, the study investigated the ability of TelePhysio to detect differences in DLS and SLS performance between people with FAI and without hip pain.

A total of 30 healthy young adults and 10 adults with diagnosed FAIS participated in the study. Healthy participants performed DLS and SLS on force plates in the laboratory and remotely in their homes using the TelePhysio smartphone application. Sway measurements were compared using the centre of pressure (CoP) and smartphone inertial sensor data. A total of 10 participants with FAI performed the squat assessments remotely. Four sway measurements in each axis (*x*, *y* and *z*) were computed from the TelePhysio inertial sensors: (i) average acceleration magnitude from the mean (aam), (ii) root-mean-square (rms) acceleration, (iii) range acceleration (*r*) and (iv) approximate entropy (apen), with lower values indicating that the movement is more regular, repetitive and predictable. Differences in TelePhysio squat sway data were compared between DLS and SLS, and between healthy and FAI adults, using analysis of variance with significance set at 0.05.

The TelePhysio aam measurements on the *x*- and *y*-axes had significant large correlations with the CoP measurements (*r* = 0.56 and *r* = 0.71, respectively). The TelePhysio aam measurements demonstrated moderate to substantial between-session reliability values of 0.73 [95% confidence interval (CI) 0.62–0.81], 0.85 (95% CI 0.79–0.91) and 0.73 (95% CI 0.62–0.82) for aam in *x*-, *y* and *z*-axes, respectively. The DLS of the FAI participants showed significantly lower aam and apen values in the mediolateral direction compared to the healthy DLS, healthy SLS and FAI SLS groups (aam = 0.13, 0.19, 0.29 and 0.29, respectively; apen = 0.33, 0.45, 0.52 and 0.48, respectively). In the anterior–posterior direction, healthy DLS showed significantly greater aam values compared to the healthy SLS, FAI DLS and FAI SLS groups (1.26, 0.61, 0.68 and 0.35, respectively).

The authors concluded that the TelePhysio app is a valid and reliable method of measuring postural control during DLS and SLS tasks. The application is capable of distinguishing performance levels between DLS and SLS tasks, and between healthy and FAI young adults. The DLS task is sufficient to distinguish the level of performance between healthy and FAI adults. This study validates the use of smartphone technology as a tele-assessment clinical tool for remote squat assessment.

## HIP EFFUSION/SYNOVITIS INFLUENCES RESULTS AFTER MULTIPLE DRILLING CORE DECOMPRESSION FOR BONE MARROW EDEMA SYNDROME OF HIP

At present, it is not known whether hip effusion/synovitis affects the therapeutic effect of multiple drilling core decompression (MDCD) in patients with bone marrow oedema syndrome of hip (BMESH). In this study, the aim of the study by Xiong *et al*. [6] was to assess hip effusion/synovitis and its relationship with the results of MDCD in patients with BMESH.

The data of undergoing arthroscopic-assisted MDCD for the treatment of BMESH with hip effusion/synovitis by one surgeon were retrospectively reviewed from the associated medical records (2016–2019). Seven patients (nine hips) participated in this study. Patients were followed up at 1, 2, 3, 6, 12 and 24 months. Data included demographics and clinical outcomes. The pre- and post-operative pain and functional outcomes were measured with the VAS, HHS, HOS-ADL, International Hip Outcome Tool-12 (iHOT-12) and range of motion (ROM).

Results: Seven patients (nine hips) were followed up. Disappearance of hip pain immediately obtained at rest after surgery. All of the seven patients returned to their former activity level at post-operative 3 months, and bone marrow oedema had disappeared on MRI. The VAS, HHS, HOS-ADL, iHOT-12 and ROM at post-operative 1 month had a significant difference compared with pre-operative. It was also statistically significant when compared with other time points. At the final follow-up, all patients had no limited ROM, which was symmetrical with the contralateral hip joint. Hip effusion/synovitis was observed in nine hips. Labral tears, cartilage fissures and loose bodies were observed in one hip, respectively. Kirschner wire tracks bleeding that occurred in one hip. No other complications occurred.

The authors thus concluded that hip effusion/synovitis could affect the clinical outcomes after MDCD in patients with BMESH. Arthroscopic procedure of hip effusion/synovitis can shorten post-operative pain relief time, disappearance time of bone marrow oedema on MRI. It can simultaneously diagnose and treat other concomitant intra-articular pathologies and be a safe operation with fewer complications.
